# Multiple myeloma with extramedullary plasmacytoma invading the skin and eyeballs following autologous stem cell transplantation: A case report

**DOI:** 10.3892/etm.2013.1246

**Published:** 2013-08-05

**Authors:** GUANGZHONG YANG, CHUANYING GENG, YANCHEN LI, AIJUN LIU, WENMING CHEN

**Affiliations:** Department of Hematology and Multiple Myeloma Research Center of Beijing, Beijing Chaoyang Hospital, Capital Medical University, Beijing 100020, P.R. China

**Keywords:** multiple myeloma, extramedullary plasmacytoma, stem cell transplantation

## Abstract

In this study, the case of a 60-year-old female patient who presented with a subcutaneous mass in the lower right limb is described. The mass was confirmed as a plasmacytoma. The patient was diagnosed with multiple myeloma (MM) λ type stage III international stage system (ISS) and received three cycles of a therapeutic PDT regimen (bortezomib, dexamethasone and thalidomide) and complete remission was achieved. Following a further two cycles of the PDT regimen, the patient proceeded to received a high-dose cyclophosphamide regimen combined with granulocyte-colony stimulating factor (G-CSF) for stem cell mobilization. Fourteen months later, the patient received a high-dose therapy supported by autologous stem cell transplantation (auto-SCT). After six months, a subcutaneous mass was identified in the left side of the patient’s neck and the mass gradually increased in size. The patient exhibited exophthalmos and loss of sight one month later. The masses in the neck and right eyelid of the patient were diagnosed as plasmacytomas. These results, combined with the results of bone marrow (BM) aspiration and protein electrophoresis with immunofixation electrophoresis revealed that the disease had relapsed. The patient received two cycles of a therapeutic CPADT regimen (cyclophosphamide, bortezomib, pharmorubicin, dexamethasone and thalidomide). The patient subsequently achieved complete remission again. The patient refused to continue receiving bortezomib and pharmorubicin for therapy and instead received four cycles of the therapeutic CTD regimen (cyclophosphamide, dexamethasone and thalidomide). Subsequently the patient received local radiotherapy for the masses in the eyes and neck. The patient remained stable after treatment following the initial relapse with a progression-free survival (PFS) time of eight months.

## Introduction

Multiple myeloma (MM) is a malignant disorder of plasma cells characterized by the proliferation of neoplastic plasma cells in the bone marrow (BM). These cells impair hematopoiesis, activate osteoclastic bone resorption and secrete a monoclonal paraprotein (M-protein) in the serum and/or urine. MM accounts for ∼1% of all human neoplasms, ∼2% of cancer mortalities and 12–15% of all cases of hematological malignancy (1).

Primary MMs are normally located in the BM, but neoplastic cells may invade other tissues and organs, such as the liver, lung, spleen, pancreas, kidney and lymph nodes (2). In the current study, we present the case of a 60-year-old female patient with MM and extramedullary plasmacytoma (EMP) that had invaded the skin and eyeballs following autologous stem cell transplantation (auto-SCT). Concomitant presentation of EMP is a poor prognostic factor, it is observed in approximately 13% of patients with multiple myeloma, and the incidence is rising (2). The study was approved by the ethics committee of Beijing Chaoyang Hospital, Capital Medical University (Beijing, China) and the informed consent was obtained from the patient

## Case report

A 60-year-old female patient presented with a painless subcutaneous mass (∼3.0×1.0 cm) in the lower right limb. The mass was confirmed as a plasmacytoma that was CD38 (+), CD138 (+), λ (+) and κ (−). The patient was diagnosed with MM λ type stage III international stage system (ISS) (3) by BM aspiration (the plasmacyte level was 21.5% in the BM) and protein electrophoresis with immunofixation electrophoresis (light chain λ in urine, 13.6 g/24 h).

The patient received three cycles of a therapeutic PDT regimen (bortezomib, dexamethasone and thalidomide) at the Beijing Chaoyang Hospital (Beijing, China) and achieved complete remission. The patient received a further two cycles of the PDT regimen and subsequently proceeded to receive a high dose cyclophosphamide (3.5 g/m^2^) regimen combined with granulocyte-colony stimulating factor (G-CSF) for stem cell mobilization and harvesting. Fourteen months later, the patient received a high-dose therapy (melphalan 200 mg/m^2^ and bortezomib) supported by auto-SCT. Following the auto-SCT, the patient remained in complete remission.

**Figure 1.**
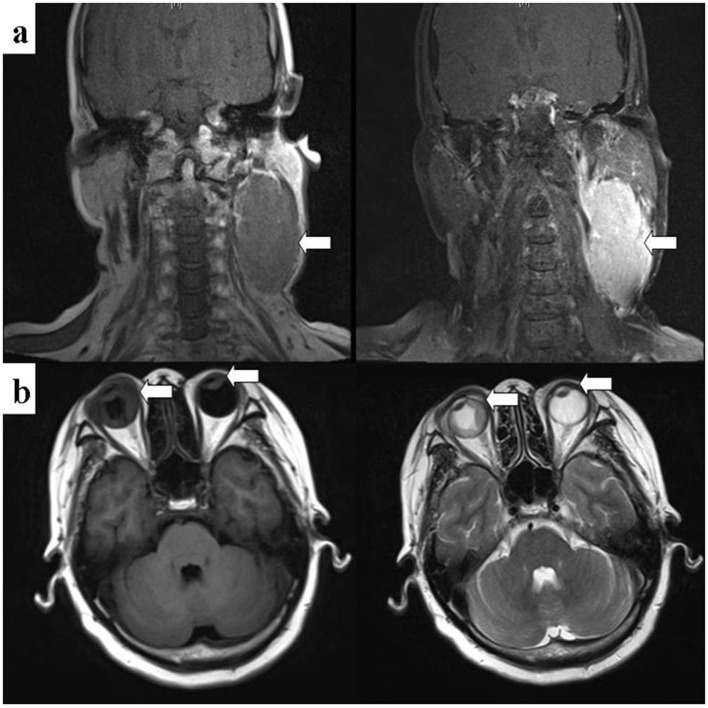
Magnetic resonance imaging (MRI) of the neck and eyeballs prior to therapy. (a) Subcutaneous mass (∼78.3×42.5 mm) in the left side of the neck. (b) The soft tissue located in the right eye socket, was enhanced markedly following the infusion of a contrast-enhancing agent. The adjacent muscles were also enhanced markedly. A 6.9×2.8 mm soft mass was observed in the left anterior chamber and was enhanced markedly following the infusion of a contrast-enhancing agent.

**Figure 2.**
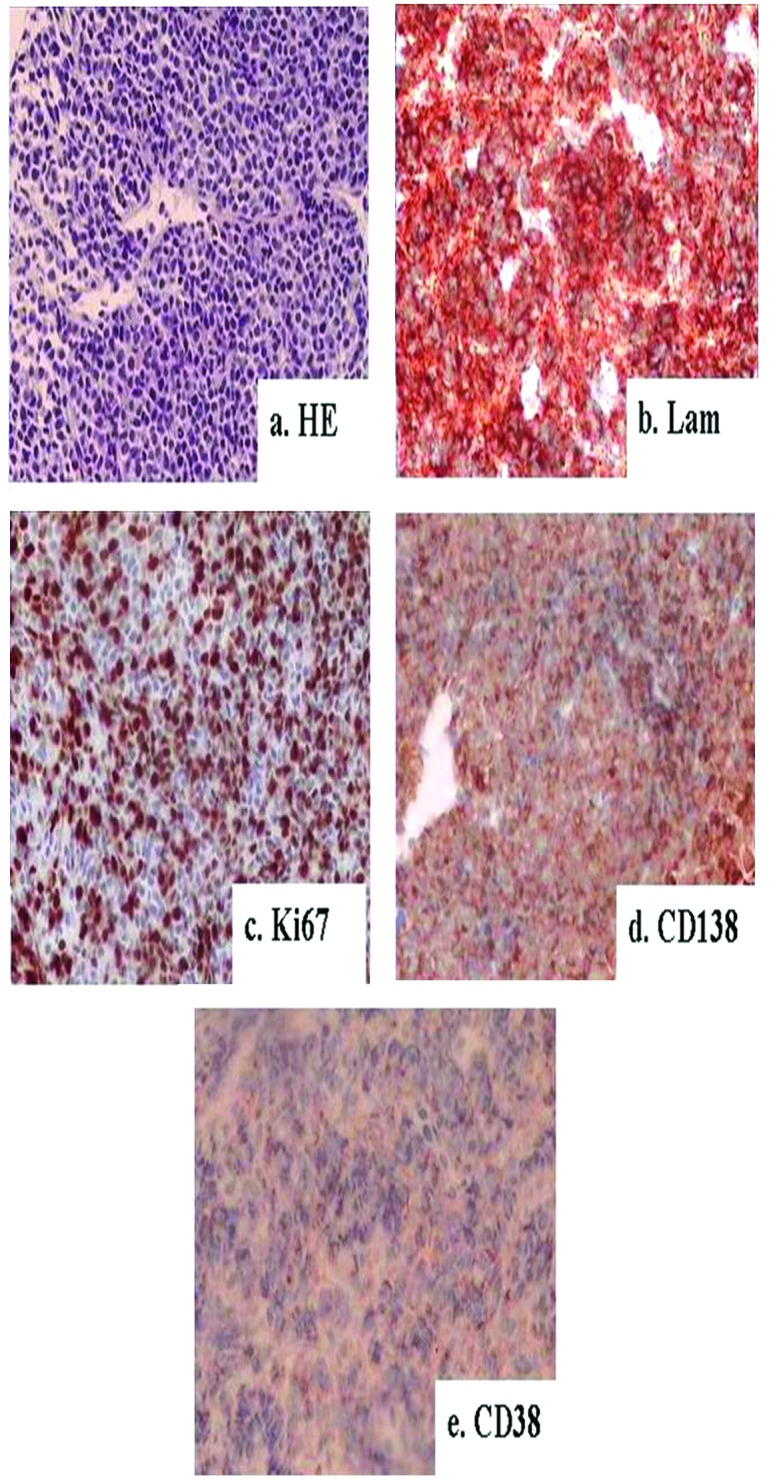
Neck plasmacytoma. (a) Histological section of the neck plasma-cytoma, hematoxylin and eosin (HE) stained. (b–e) CD38, CD138 and light chain λ (Lam) were positive by immunohistochemical staining; the positive ratio of Ki-67 was >60%. Magnification, ×400.

**Figure 3.**
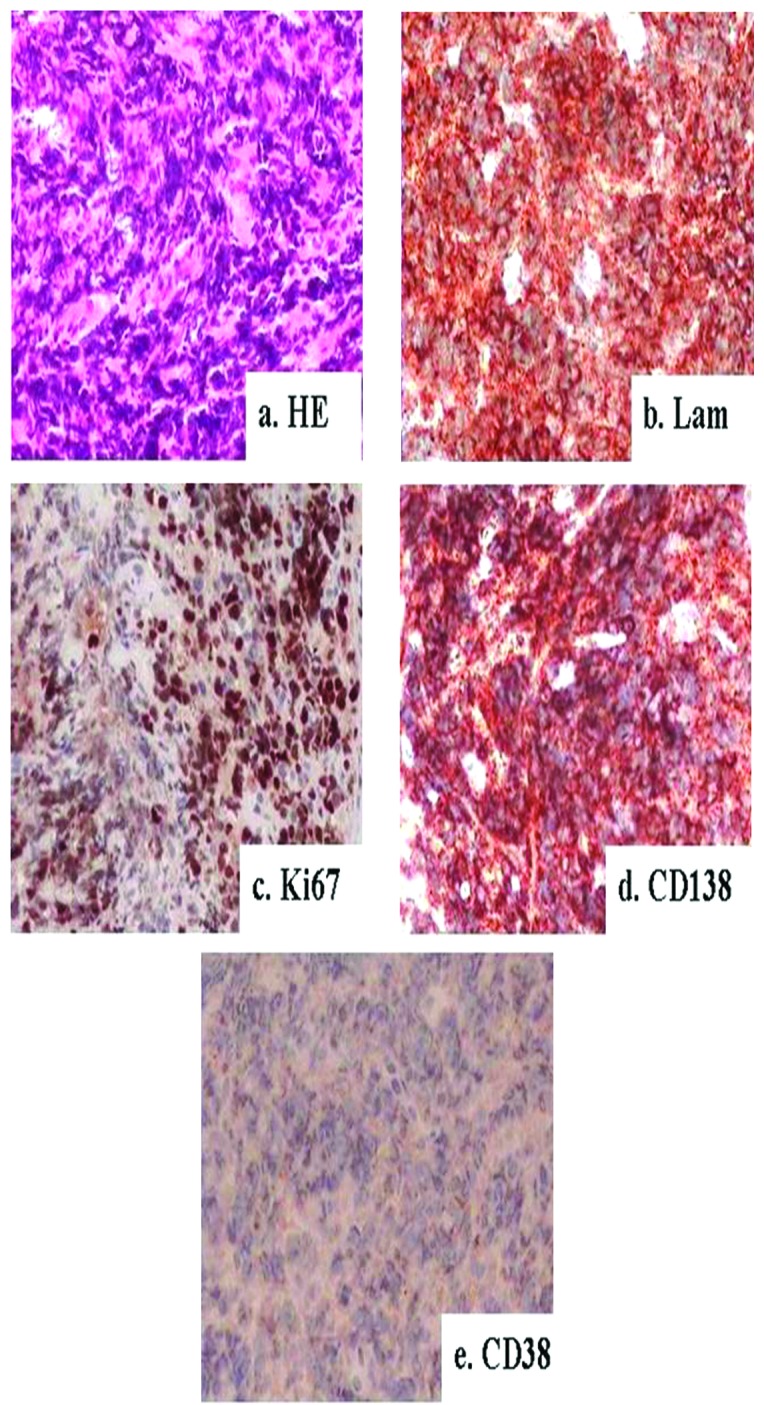
Right eyelid plasmacytoma. (a) Histological section of the right eyelid plasmacytoma, hematoxylin and eosin (HE) stained. (b–e) CD38, CD138 and light chain λ (Lam) were positive by immunohistochemical staining; the positive ratio of Ki-67 was ∼80%. Magnification, ×400.

**Figure 4.**
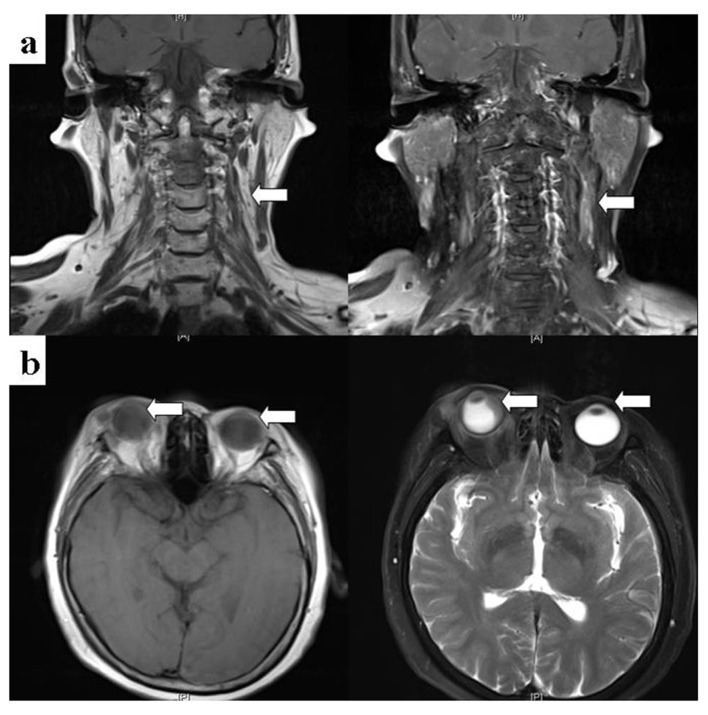
Magnetic resonance imaging (MRI) of the neck and eyeballs following two cycles of therapy. (a) Flake shadow, located in the left of the neck, was markedly enhanced following the infusion of a contrast-enhancing agent. The mass was reduced in size following treatment. (b) Both eye sockets thickened and were enhanced homogeneously. The range that was invaded by multiple myeloma reduced by approximately 60%.

Six months later, a painless subcutaneous mass (∼1.5×1.0 cm) was identified in the left side of the patient’s neck and the mass gradually increased in size. One month later, the patient exhibited exophthalmos and loss of sight. We located a mass ∼7.8×4.2 cm in the left side of the patient’s neck and observed that the patient’s eyeballs were abnormal by using magnetic resonance imaging (MRI). A 6.9×2.8 mm soft mass was observed in the left anterior chamber and was enhanced markedly following the infusion of a contrast-enhancing agent (Fig. 1). Pathology results from the masses in the neck and right eyelid revealed that the masses were plasmacytomas (Figs. 2 and 3). The pathology results and MRI combined with the results of BM aspiration (plasmacyte levels of 11.0% in the BM) and protein electrophoresis with immunofixation electrophoresis (light chain λ in urine, 1.2 g/24 h) revealed that the disease had relapsed. The patient received two cycles of the therapeutic CPADT regimen (cyclophosphamide, bortezomib, pharmorubicin, dexamethasone and thalidomide). The response to the treatment was assessed by BM aspiration and protein electrophoresis with immunofixation electrophoresis. The patient achieved complete remission again with negative immunofixation electrophoresis and plasmacyte levels of 1% in the BM. Furthermore, the patient’s exophthalmos improved, the mass in the patient’s neck was markedly reduced in size and the mass in the eyeball ring was also smaller (Fig. 4). However, the patient’s visual acuity did not improve. The patient refused to continue receiving bortezomib and pharmorubicin for therapy due to severe BM inhibition and infection. The patient continued to receive four cycles of the therapeutic CTD regimen (cyclophosphamide, dexamethasone and thalidomide). Subsequently, the patient received local radiotherapy for the neck and eyes. The patient remained stable since the treatment that followed the initial relapse with a progression-free survival (PFS) time of eight months.

## Discussion

EMP may occur either at diagnosis or during the course of MM. In a longitudinal study, 13% of MM patients had EMP (7% at diagnosis and 6% during follow-up) (2). A high incidence of EM relapses has been reported following autologous and allogeneic SCT (4,5,6). The patient in the current study exhibited EM and medullory progression following auto-SCT. However, the incidence of EMP relapse is not higher in patients receiving high-dose therapy (HDT) compared with other treatments (2). The widespread use of more sensitive imaging techniques such as computed tomography (CT) and MRI may partially explain the phenomena.

The patient in the current study was diagnosed with two plasmacytomas, in the eyeball and the neck. The majority of MM patients with EMP have a single plasmacytoma. In a previous study, in 85% of cases the sites affected were the soft tissues surrounding the axial skeleton; plasmacytomas of the lymph nodes, liver, kidney, airways, skin and breast, accounted for 15% (2).

The patient in the current study was identified as having low M-protein levels during disease progression, but the positive rate of Ki-67 in EMP was very high. However, the lactate dehydrogenase (LDH) level of the patient was normal. Notably, Dawson *et al* (7) reported three MM patients who underwent EM relapse associated with a shift in the secretion of intact immunoglobulins to free light chains, known as the ‘light chain escape from plateau phase’ (LEPP). The syndrome was characterized by multiple EM sites of relapse, plasmablastic features, renal failure, high LDH and β2-microglobulin levels and an aggressive course of clinical treatment. The authors hypothesized that LEPP results from clonal selection and the expansion of precursors that have lost the ability to secrete intact immunoglobulins while acquiring stromal independence and the ability to spread outside the BM (7). Furthermore, they indicated that LEPP may be derived from the effect of novel agents, including bortezomib and lenalidomide, on the BM microenvironment since LEPP occurred following novel therapies such as thalidomide or lenalidomide. Other authors have not identified a relationship between the EM spread of disease and prior exposure to novel agents (2).

In the current study, a combination therapy, including thalidomide and bortezomib, was administered. Following two cycles of therapy, the patient experienced a marked remission. The introduction of thalidomide, bortezomib, and lenalidomide has expanded the therapeutic armamentarium for MM (8–10). However, to date no studies have focused on the treatment of MM patients with EMP. Certain studies have indicated that bortezomib is more promising in this environment (11,12). Radiotherapy is normally associated as a systemic treatment with chemotherapy or other novel agents.

In a study of 19 patients with EMP and extraosseous MM, the disease was observed to follow an aggressive course, with a median overall survival (OS) of 15 months (13). Terpos *et al* (5) noted that isolated EMP relapses following HDT were almost invariably followed by systemic progression with short OS. However, in another study of 78 patients who relapsed following autologous or allogeneic SCT, the outcome of patients with EMP or medullary relapse was not significantly different (4). To the best of our knowledge, data concerning the prognosis of EMP in MM are limited and controversial since certain studies show that the patients with EMP and extraosseous MM had a poor prognosis, but others show that the outcome of patients with EMP or medullary relapse was not significantly different (4,5,13).

In conclusion, the patient with extramedullary plasma-cytoma invading skin and eyeballs following autologous stem cell transplantation in the present study had a favorable response after combination therapy with bortezomib. Such patients require clinical studies with novel treatment strategies for a better prognosis.
